# Editorial Notes, News and Miscellany

**Published:** 1913-12

**Authors:** 


### Editorial Notes, News and Miscellany.

Death of Dr. Hudson of Milano. We are pained to announce the death of our old friend, a most estimable gentleman and physician. He died at his home in Milano, October 17th and was buried at his old Kentucky home, Nuckols, Kentucky. We clip the following tribute to his memory from his local paper. Dr. Hudson was a graduate of Memphis Medical College in 1886, and of Medical Department, University of Louisville, 1889:

### Obituary.

IN MEMORY OF DR. J. W. HUDSON.

Dr. John William Hudson was born in McLane county, Kentucky, where the town of Nuckols now stands, on December 21, 1845. His mother was a daughter of a very prominent physician of that time, Dr. Jones. At the age of 22 years, Dr. Hudson was married to Miss Luvenia Atherton, daughter of John G. Atherton, who, it will be recalled by a few, died February 11, 1913, at the age of 92 years. To this union was born two children, Beulah, who died at the age of 18 months and Miss Claudie who survives him. His wife died in 1880 after a lingering illness.

In 1882 he was married to Miss Fannie Stroud of Central City, Kentucky, who now survives him. Moving to Milano that year he continued to cast his lot with the people of this country, until called to his home above on October 17, 1913.

While Milano was his home, the Doctor had many ties that bound him to the place and State of his birth. The old home in which he was born still stands in Nuckols and the Doctor enjoyed very much taking trips back to the scenes of his boyhood days. It will be remembered that he had only been back from such a visit about seven weeks before his death. His wife, his daughter, Mr. J. E. Kelly and Mr. Baxter Stroud, Mrs. Hudson's brother, accompanied the remains back to Kentucky where he was laid to rest Tuesday, October 21st, in the Buck Creek Cemetery at Nuckols, besides his first wife and child.

The Doctor was an earnest and devout Christian, having joined the Buck Creek Baptist Church in Kentucky when 19 years of age, from which place his membership was moved to our Milano Baptist Church shortly after he moved here. He was for years superintendent of our Sunday school and truly loved his church.

He was a member of several fraternal organizations, being a charter member of the Knights of Pythias Lodge at Rockdale, a member of the Knights of Honor of the same place and an active member of the Masonic and Odd Fellow Lodges of this place. He was a man who will be greatly missed in the councils of his orders, he being a faithful and strict adherent to his obligations and to the tenets and teachings of his lodges.

As a man, Milano's loss is inestimable. No greater friend to the poor and needy lived among us. Always ready at a call to visit the sick and do all in his power to relieve their suffering, thousands of times expecting no pay, getting no pay nor asking none.

There is a vacancy in our midst, a voice in our walks of life has been silenced and sadness has found place in our hearts. Memory holds clear and fond affections cherishes the soul we knew. Once, only once, but surely once, will come to every one of us the call that takes a soul from this earth into the great beyond. We cannot know why one rather than another has been chosen. One greater and far more wise has full control of this great question. It is our duty, with the best grace we can command, to bow submissively to the will of God. May this tie bring us into that sympathy and harmony of spirit, that is ever found in the union of memories, of affections of sorrows and aspirations. His spirit has returned to the God who gave it and his memory we shall always cherish in our hearts.

MARRIED at Austin November 5 (ult.), Dr. A. F. Beverly to Miss Bessie Eilers, daughter of Mr. and Mrs. A. J. Eilers, all of Austin.

---

MARRIED at Austin November 19, Mr. Frank Domschke, President of the Von Boeckmann-Jones Printing Co., to Miss Sophie Matthiesen, one of the prettiest girls in Texas. Frank is surely to be congratulated.

---

The Sixth District Medical Association was organized in Corpus Christi, October 14, 15, 16. Dr. F. U. Painter was elected president; Dr. T. J. Turpin, vice-president and Dr. L. J. Manhoff secretary and treasurer. The membership is composed of some eighty odd young, vigorous and enthusiastic physicians, which insures a long-lived and a successful organization. Many interesting papers were read and discussed in a manner that showed that the membership of this body is abreast with medical progress and its theories of today. A very hearty and cordial comradeship was evident on all sides. The social features consisted of an excursion, aboard the power boat Japonica, to Port Aransas, where one of the most elaborate and bountiful fish feasts ever spread in these parts was enjoyed by the association and its guests; and of an excellent and most enjoyable banquet at the Nueces hotel, in all of which the wives participated, including post-prandial speech making participated in by the ladies. BIBB.

